# Obesity-associated microbiota contributes to mucus layer defects in genetically obese mice

**DOI:** 10.1074/jbc.RA120.015771

**Published:** 2020-09-08

**Authors:** Bjoern O. Schroeder, George M. H. Birchenough, Meenakshi Pradhan, Elisabeth E. L. Nyström, Marcus Henricsson, Gunnar C. Hansson, Fredrik Bäckhed

**Affiliations:** 1 Wallenberg Laboratory, Department of Molecular and Clinical Medicine, Institute of Medicine, University of Gothenburg, Gothenburg, Sweden; 2 Laboratory for Molecular Infection Medicine Sweden (MIMS), Department of Molecular Biology, Umeå University, Umeå, Sweden; 3 Department of Medical BiochemistryInstitute of Biomedicine, University of Gothenburg, Gothenburg, Sweden; 4 Novo Nordisk Foundation Center for Basic Metabolic Research, Faculty of Health Sciences, University of Copenhagen, Copenhagen, Denmark; 5 Department of Clinical Physiology, Region Västra Götaland, Sahlgrenska University Hospital, Gothenburg, Sweden

**Keywords:** barrier dysfunction, gut microbiota, host defense, intestinal epithelium, metabolic disease, mucus, mucosal immunology, obesity, microbiome, metabolism

## Abstract

The intestinal mucus layer is a physical barrier separating the tremendous number of gut bacteria from the host epithelium. Defects in the mucus layer have been linked to metabolic diseases, but previous studies predominantly investigated mucus function during high-caloric/low-fiber dietary interventions, thus making it difficult to separate effects mediated directly through diet quality from potential obesity-dependent effects. As such, we decided to examine mucus function in mouse models with metabolic disease to distinguish these factors. Here we show that, in contrast to their lean littermates, genetically obese (ob/ob) mice have a defective inner colonic mucus layer that is characterized by increased penetrability and a reduced mucus growth rate. Exploiting the coprophagic behavior of mice, we next co-housed ob/ob and lean mice to investigate if the gut microbiota contributed to these phenotypes. Co-housing rescued the defect of the mucus growth rate, whereas mucus penetrability displayed an intermediate phenotype in both mouse groups. Of note, non-obese diabetic mice with high blood glucose levels displayed a healthy colonic mucus barrier, indicating that the mucus defect is obesity- rather than glucose-mediated. Thus, our data suggest that the gut microbiota community of obesity-prone mice may regulate obesity-associated defects in the colonic mucosal barrier, even in the presence of dietary fiber.

The mammalian intestine harbors a complex microbial ecosystem where bacterial densities can reach up to 10^11^ cells/g of content and thus challenge the mucosal defense systems (1). To prevent the microbiota from directly accessing the mucosal lining, the intestine is covered with a layer of mucus as a first line of defense. This gel-like network, which in the intestine is mainly composed of the highly glycosylated mucin protein MUC2, physically separates the gut bacteria from the intestinal epithelium (2).

Recently, it has become evident that formation of the mucus layer is not entirely a host-controlled process and that the presence of gut bacteria is required to fully mature this defense. Remarkably, the composition of the gut microbiota determines the success of mucus maturation (3), and diet-mediated alterations of the gut microbial community can have distinct effects on mucus function. Specifically, when mice are fed a diet devoid of complex microbiota-accessible carbohydrate, microbial glycan degradation can lead to deterioration of the mucus barrier and consequently allow gut bacteria to approach or even translocate the mucosa (4–7).

Microbial translocation across the intestinal barrier has commonly been associated with infection, but recently there has been growing interest in translocation of bacterial products in the context of metabolic diseases. As such, it was shown that gut-derived endotoxin can initiate obesity and insulin resistance in mice (8, 9) and humans with obesity (10) and hyperglycemia (11) have a penetrable mucosal barrier. Moreover, mice that are deficient in the intestinal bacterial recognition receptor Toll-like receptor 5 developed microbiota-dependent metabolic impairments (12) and mice that lack the bacteria-aggregating mucus protein ZG16 (zymogen granule protein 16) displayed a microbiota-dependent increase in abdominal fat pad mass (13).

Studies investigating metabolic phenotypes frequently use high-caloric diets to induce metabolic impairments, making it difficult to disentangle the contribution of diet quality and metabolic factors. As we previously observed that diet-induced obesity in mice led to microbiota-dependent defects of the inner colonic mucus layer (7), we now aimed to disentangle the interaction between gut bacteria, host obesity, and mucus function independent of dietary composition. Therefore, we analyzed mucus properties in genetically obese mice that are deficient in the satiety hormone leptin (ob/ob mice), and thus become severely obese through hyperphagia.

## Results

**Figure 1.**
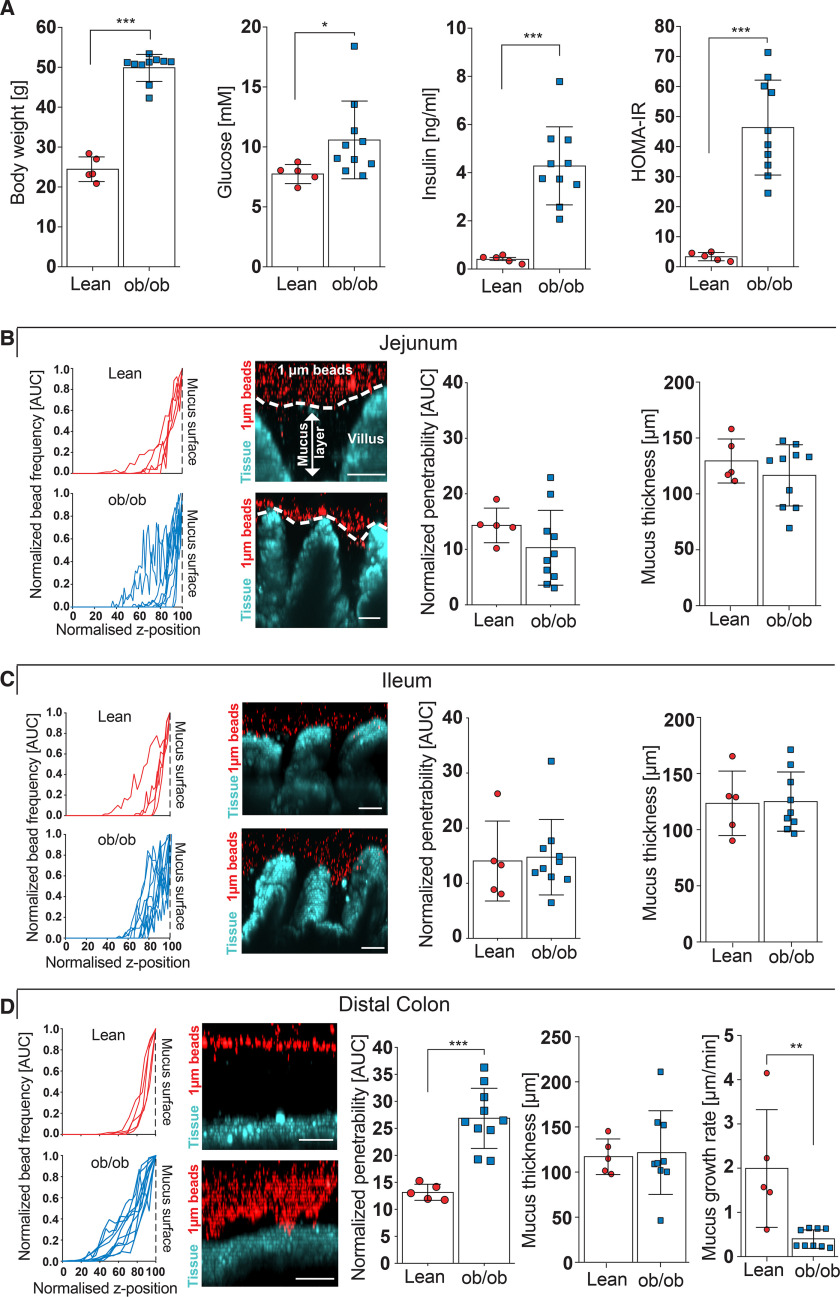
**Intestinal mucus function in genetically obese (ob/ob) mice.** *A*, body weight, fasting blood glucose, fasting insulin concentration, and HOMA IR in littermate-controlled lean and genetically obese (ob/ob) mice (*n* = 5–10 mice per genotype). *B–D*, mucus properties of the mucus layer in the jejunum (*B*), ileum (*C*), and distal colon (*D*). From left to right: the position of fluorescent 1-μm beads, obtained from confocal z-stacks were used to determine normalized penetrability of the mucus layer. *Turquoise*, intestinal tissue; *red*, 1 μm bacteria-sized beads. Thickness and growth rate (*D*) of the mucus layer was measured *ex vivo* with a micromanipulator by measuring the distance between black 10-μm beads and the epithelial surface (*n* = 5–10 mice per genotype). *Scale bar* = 50 μm. Data in *A–D* are presented as mean ± S.D. *, *p* ≤ 0.05, **, *p* ≤ 0.01; ***, *p* ≤ 0.001 (Mann-Whitney *U* test). For the normalized bead frequency (*left*), the median bead frequency distribution is shown for each mouse.

To investigate mucus function during obesity not confounded by diet, we compared genetically obese (ob/ob) leptin-deficient mice with their lean, separately housed littermates that were all fed a fiber-containing control chow diet. As expected, 16-week–old ob/ob mice weighed almost twice as much as their lean littermates (Fig. 1*A*, *p* < 0.001) and fasting blood glucose (*p* = 0.021) and insulin levels (*p* < 0.001) were increased in ob/ob mice, resulting in a higher homeostatic model assessment of insulin resistance (HOMA IR, *p* < 0.001), which indicates increased insulin resistance.

Mucus in the small intestine has been described to be loose and penetrable (14). However, by analyzing mucus function in the jejunum (Fig. 1*B*) and ileum (Fig. 1*C*) by an *ex vivo* explant method (15), we observed predominantly impenetrable mucus between the intestinal villi, a phenotype that was similar between lean and obese mice. In contrast to the small intestinal mucus, we observed strongly increased penetrability of the inner colonic mucus layer in genetically obese mice when compared with their lean littermates (Fig. 1*D*). Moreover, whereas we did not observe any difference in initial mucus thickness between the two genotypes, mucus growth rate, which is thought to be a combination of mucus secretion and tightly regulated proteolytic processing (16), was markedly reduced in the ob/ob mice. Accordingly, genetically obese mice have a penetrable inner colonic mucus layer with a slow mucus growth rate, which is a barrier defect that can also be caused by Western-style diet (WSD)-mediated alterations of the gut microbiota (7).

**Figure 2.**
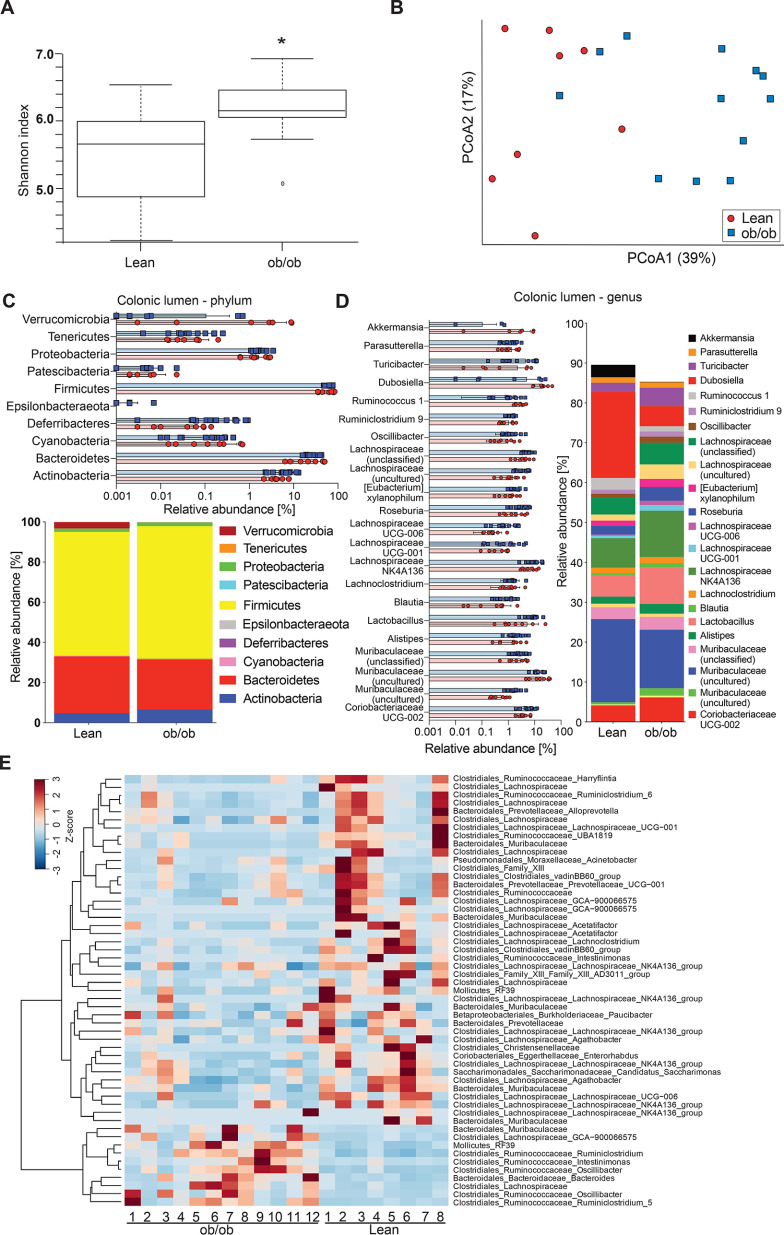
**Gut microbiota analyses in the colonic lumen of ob/ob mice.** *A* and *B*, α*-*diversity (Shannon index, A) and β-diversity (Bray-Curtis dissimilarity matrix), *B*, for the luminal microbial community (*n* = 8-12 mice per genotype). Statistically significant difference was tested with Kruskal-Wallis test with: *, *p* ≤ 0.05 for α-diversity (*A*) and with PERMANOVA and 999 permutations for β-diversity (*B*). *C* and *D*, relative abundance of microbial taxa on phylum (*C*) and genus level (*D*). In the colored bar representation, microbial taxa with an abundance >1% are shown. *E*, heatmap showing the differentially abundant ASVs in the colonic content of ob/ob and lean mice. Heatmap is based on hierarchical clustering of the differentially abundant ASVs calculated using DESeq2 (Wald test, FDR < 0.05).

As the gut microbiota has been shown to be an essential modulator of mucus function (3, 7, 17), we therefore investigated gut microbiota composition in the colons of ob/ob and lean mice. α-Diversity, as measured by Shannon index, was slightly higher in ob/ob mice (*p* = 0.045; Fig. 2*A*) compared with the lean controls. The β-diversity revealed a significant clustering according to genotype, as shown in the principal coordinate analysis using Bray-Cutis dissimilarity matrix (*p* = 0.002; Fig. 2*B*) and in the principal component analysis plots using unweighted (*p* = 0.003; Fig. S1*A*) and weighted UniFrac (*p* = 0.008; Fig. S1*B*) distance matrices. Because unweighted UniFrac analyses produced greater separation than weighted UniFrac analyses, lean and obese mice appeared to mainly differ in low abundant bacteria. Only minor differences in phylum (Fig. 2*C*) or genus level (Fig. 2*D*) were observed. For example, were relative abundance of Akkermansia (Verrucomicrobia) and Dubosiella (Firmicutes) reduced in the colon of ob/ob mice.

To further investigate the microbial profile of the colonic microbiota we used DESeq2 to identify differentially abundant amplicon sequence variants (ASVs). We observed 53 ASVs that were discriminatory between the colonic luminal microbiota of lean and obese mice (43 ASVs depleted and 10 ASVs enriched in obese mice) (Fig. 2*E*). Of note, we identified already previously that the Muribaculaceae family (previously called S24-7) was associated with intact function of the inner colonic mucus layer (18). However, the Muribaculaceae and Lachnospiraceae families (specifically its genus GCA−900066575) were represented by distinct ASVs that were both discriminatory for lean and obese mice, which supports our interpretation of the β-diversity analysis that the difference between the lean and the obese mice is based on closely related microbial taxa.

An altered microbial community can result in differences in metabolite production, including short-chain fatty acids (SCFAs)(19), which have been described to modulate mucus expression (20, 21). Furthermore, butyrate is the preferred energy source used by colonocytes (22), including goblet cells, and accordingly changes in the SCFA profile may have profound effects on physiology. We therefore analyzed the concentration of SCFAs as well as the organic acids lactate, succinate, isobutyrate, and isovalerate in the cecum of the mice (Fig. S1*C*). However, the levels of these fermentation products were similar in lean and obese mice and did not correlate with mucus growth rate (Fig. S1*D*) or mucus penetrability (Fig. S1*E*). In agreement with our previous findings on WSD mice (7) we concluded that SCFAs are not the major driver for the mucus defects in these mouse models.

**Figure 3.**
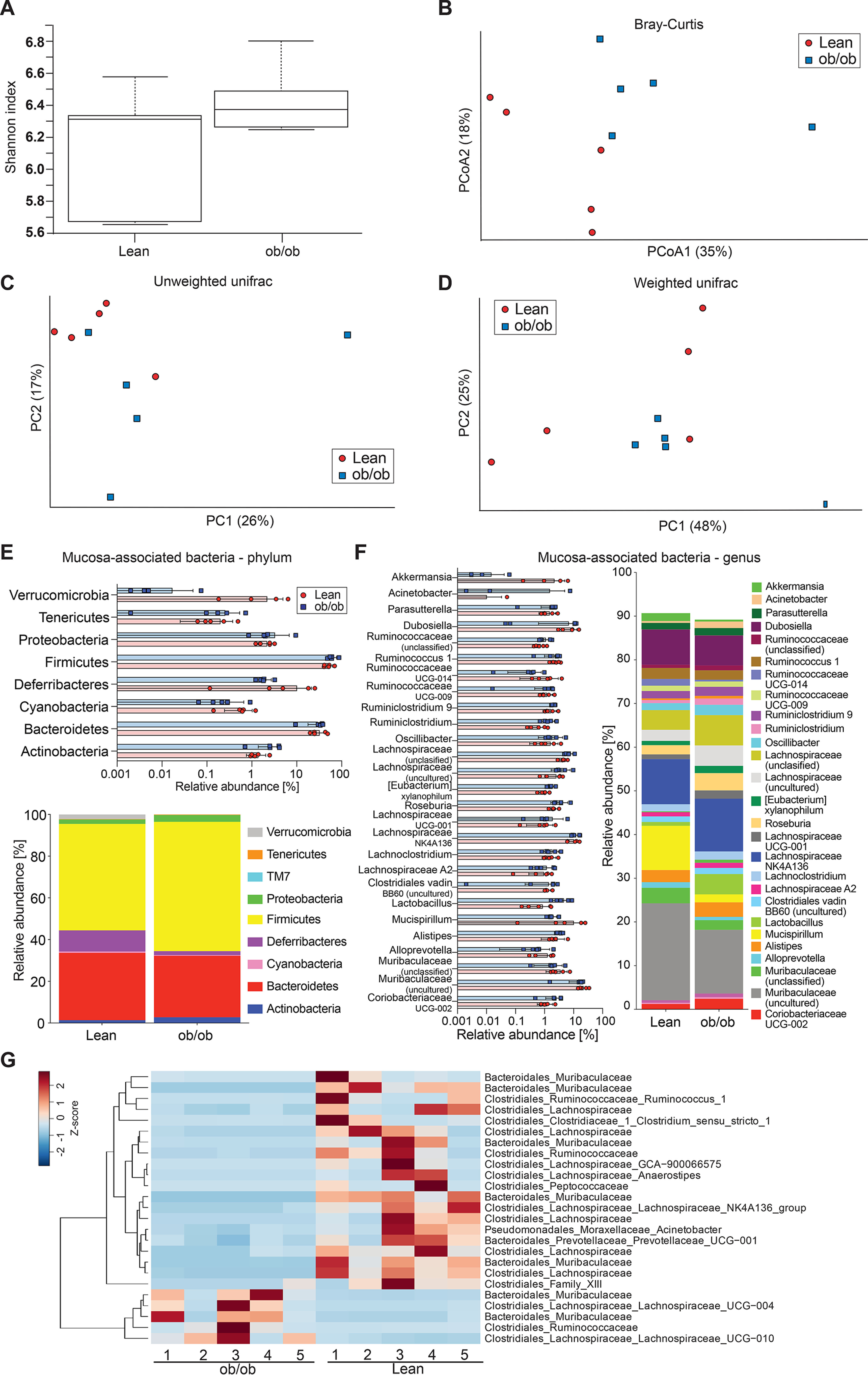
**Gut microbiota analyses at the colonic mucosal surface.** *A–D*, α-diversity (Shannon index, *A*) and β-diversity (Bray-Curtis dissimilarity matrix, *B*; unweighted (*C*) and weighted (*D*) UniFrac distance matrices) for the mucosa-associated microbial community of genotype-separated lean and ob/ob mice (*n* = 5 mice per genotype). Statistically significant difference was tested with Kruskal-Wallis test with: *, *p* ≤ 0.05 for α-diversity (*A*) and with PERMANOVA and 999 permutations for β-diversity (*B–D*). *E* and *F*, relative abundance of microbial taxa on phylum (*E*) and genus level (*F*). In the colored bar representation, microbial taxa with an abundance >1% are shown. *G*, heatmap showing the differentially abundant ASVs at the colonic mucosa of genotype-separated ob/ob and lean mice. Heatmap is based on hierarchical clustering of the differentially abundant ASVs calculated using DESeq2 (Wald test, FDR < 0.05).

Mucus properties are likely influenced by the bacteria that are in close proximity to the epithelium, and we thus also investigated the microbial composition at the colonic mucosa (Fig. 3). Here, we did not detect any difference in α-diversity (Fig. 3*A*, *p* = 0.35), whereas β-diversity analysis showed significant genotype-dependent clustering based on principal coordinate analysis using Bray-Cutis dissimilarity matrix (Fig. 3*B*; *p* = 0.009) and principal component analysis using unweighted UniFrac (Fig. 3*C*; *p* = 0.049), but not when using weighted UniFrac analysis (Fig. 3*D*; *p* = 0.167). On the phylum level, the composition at the mucosa resembles that of the lumen, except for higher abundance of Deferribacteres in the mucus niche, which had lower relative abundance in obese compared with lean mice (Fig. 3*E*). Moreover, discriminatory taxa were dominated by mostly unclassified members of the Muribaculaceae, Ruminococcaceae, and Lachnospiraceae families, which were discriminatory for both groups (Fig. 3, *F* and *G*).

**Figure 4.**
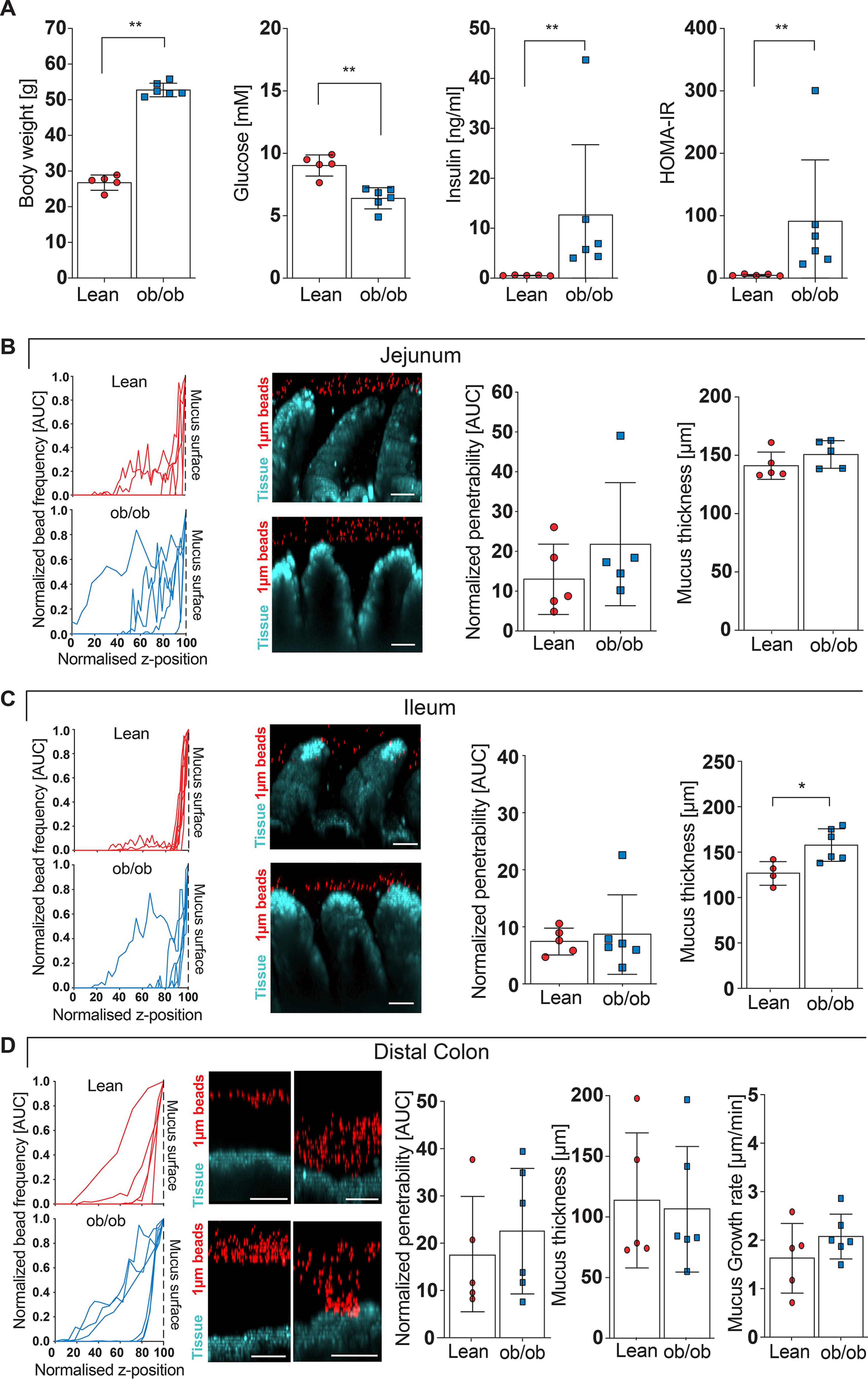
**Intestinal mucus function in co-housed lean and ob/ob mice.** *A*, body weight, fasting blood glucose, fasting insulin concentration, and HOMA IR in littermate-controlled lean and genetically obese (ob/ob) mice (*n* = 5-6 mice per genotype). *B–D*, mucus properties of the mucus layer in the jejunum (*B*), ileum (*C*), and distal colon (*D*). From left to right: the position of fluorescent 1-μm beads, obtained from confocal z-stacks were used to determine normalized penetrability of the mucus layer. *Turquoise*, intestinal tissue; *red*, 1-μm bacteria-sized beads. Thickness and growth rate (*D*) of the mucus layer was measured *ex vivo* with a micromanipulator by measuring the distance between black 10-μm beads and the epithelial surface (*n* = 4-6 mice per genotype). *Scale bar* = 50 μm. Data in *A–D* are presented as mean ± S.D. *, *p* ≤ 0.05; **, *p* ≤ 0.01 (Mann-Whitney *U* test). For the normalized bead frequency (*left*), for the normalized bead frequency, the median bead frequency distribution is shown for each mouse.

Based on the finding that genetically obese mice have a mucus defect and altered gut microbiota composition compared with their lean littermates we raised the hypothesis that the “obese” microbiota may contribute to altered mucus properties. We thus co-housed lean and obese littermates instead of separating them after weaning, allowing microbiota transfer through the coprophagic behavior of the animals. Body weight (*p* = 0.004), insulin concentration (*p* = 0.004), and HOMA IR (*p* = 0.004) were similarly different after the co-housing as compared with the genotype-separated animals (Fig. 1*A*), whereas fasting blood glucose concentration in the ob/ob mice was lower as in their lean littermates (*p* = 0.004), suggesting that the glucose phenotype may also be attributed to differences in the microbiota (Fig. 4A).

**Figure 5.**
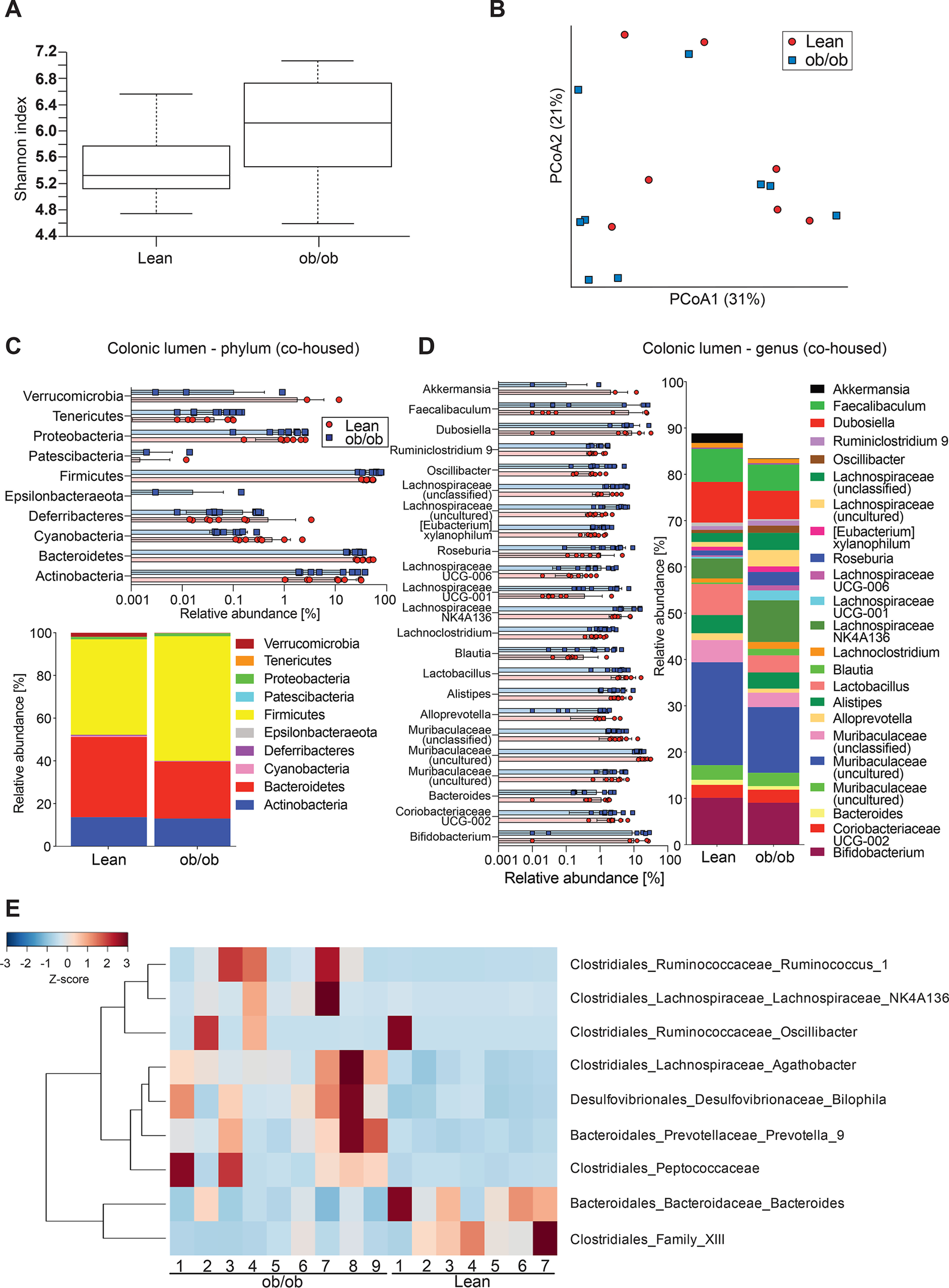
**Gut microbiota analyses in the colonic lumen of co-housed ob/ob and lean mice.** *A* and *B*, α-diversity (Shannon index, *A*) and β-diversity (Bray-Curtis dissimilarity matrix, *B*) for the luminal microbial community (*n* = 7-9 mice per genotype). Statistically significant difference was tested with Kruskal-Wallis test with: *, *p* ≤ 0.05 for α-diversity (*A*) and with PERMANOVA and 999 permutations for β-diversity (*B*). *C* and *D*, relative abundance of microbial taxa on phylum (*C*) and genus level (*D*). In the colored bar representation, microbial taxa with an abundance >1% are shown. *E*, heatmap showing the differentially abundant ASVs in the colonic content of co-housed ob/ob and lean mice. Heatmap is based on hierarchical clustering of the differentially abundant ASVs calculated using DESeq2 (Wald test, FDR < 0.05).

When analyzing mucus function in the co-housed mice, again no difference in jejunal (Fig. 4*B*) or ileal (Fig. 4*C*) mucus penetrability was observed, whereas the ob/ob mice displayed a slight increase in mucus thickness in their ileum (Fig. 4*C*). In the distal colon, however, co-housing abrogated the previously observed differences in penetrability and growth rate of the inner mucus layer (Fig. 4*D*), indicating that the gut microbiota causes the mucus phenotype in obese mice. As expected, co-housing normalized the microbiota and no significant difference between the genotypes was observed (α-diversity shown in Shannon index *p* = 0.12 (Fig. 5*A*), β-diversity shown in Bray-Curtis dissimilarity, *p* = 0.319, unweighted UniFrac, *p* = 0.564, and weighted UniFrac, *p* = 0.089) (Fig. 5*B* and Fig. S2, *A* and *B*). Instead, we identified kinship as a significant contributor to the microbiota diversity (Bray-Curtis dissimilarity, *p* = 0.001; unweighted UniFrac, *p* = 0.001; weighted UniFrac: *p* = 0.003), thus supporting a successful microbiota transfer within the co-housed litters. Consistently, we did not observe any major differences between the ob/ob and lean mice on phylum and genus level (Fig. 5, *C* and *D*) and found that only 9 ASVs discriminated between the lean and the ob/ob mice (Fig. 5*E*), which is in contrast to the 53 ASVs identified between the genotype-separated mice (Fig. 2*E*). Among them, two Lachnospiraceae (Lachnospiraceae_NK4A136 and Agathobacter) and two Ruminococcaceae (Ruminococcus_1 and Oscillibacter) were enriched at the mucosa of ob/ob mice, whereas Bacteroides and the Clostridiales_Family_XIII were enriched at the mucosa of lean mice. Furthermore, we did not observe any differences in SCFA levels (Fig. S2*C*).

**Figure 6.**
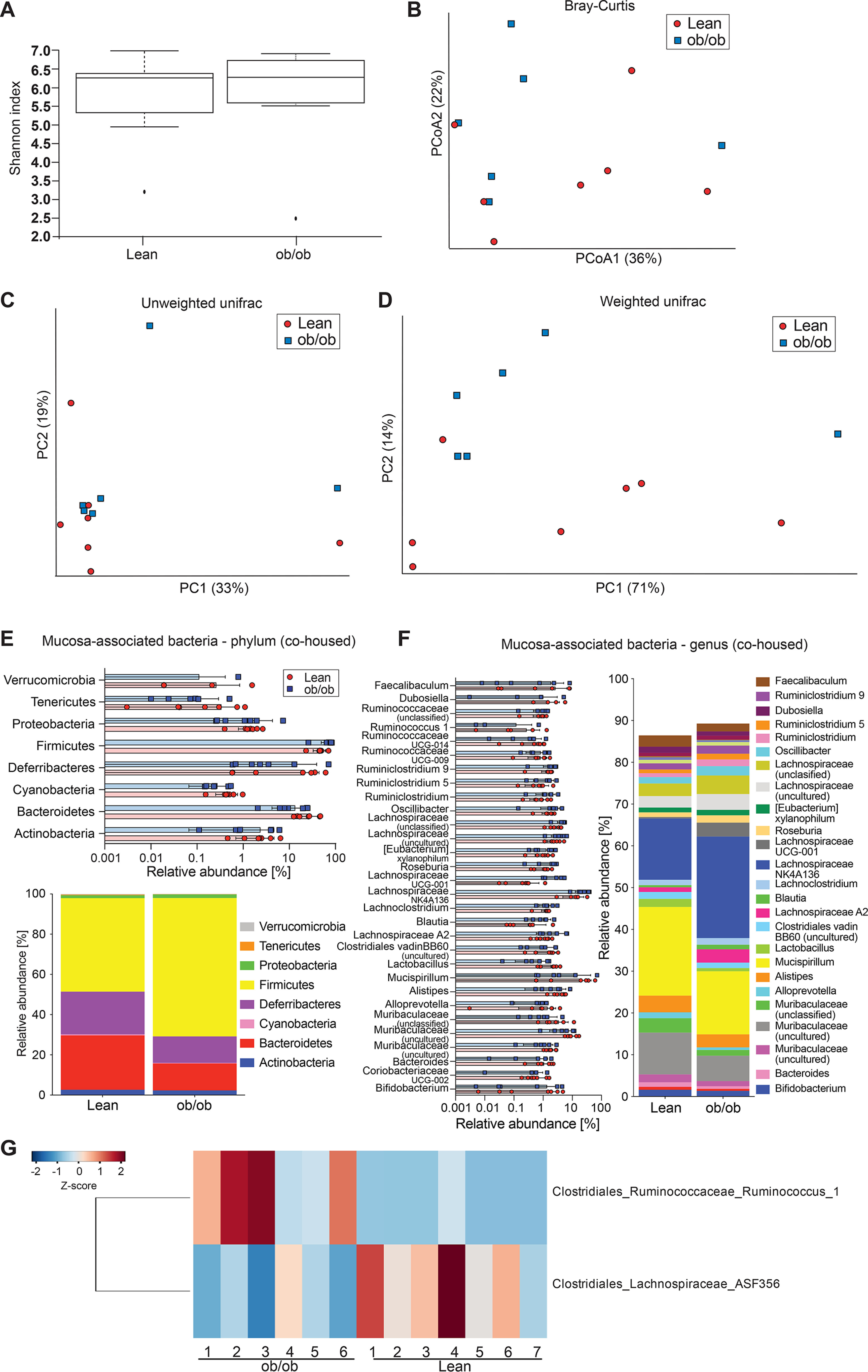
**Gut microbiota analyses at the colonic mucosal surface of co-housed lean and ob/ob mice.** *A–D*, α-diversity (Shannon index, *A*) and β-diversity (Bray-Curtis dissimilarity matrix, *B*; unweighted (*C*) and weighted (*D*) UniFrac distance matrices) for the mucosa-associated microbial community of co-housed lean and ob/ob mice (*n* = 6-7 mice per genotype). Statistically significant difference was tested with Kruskal-Wallis test with: *, *p* ≤ 0.05 for α-diversity (*A*) and with PERMANOVA and 999 permutations for β-diversity (*B–D*). *E* and *F*, relative abundance of microbial taxa on phylum (*E*) and genus level (*F*). In the colored bar representation, microbial taxa with an abundance >1% are shown. *G*, heatmap showing the differentially abundant ASVs at the colonic mucosa of co-housed ob/ob and lean mice. Heatmap is based on hierarchical clustering of the differentially abundant ASVs calculated using DESeq2 (Wald test, FDR < 0.05).

At the mucosa, the microbial diversity was not different between the genotypes anymore (Fig. 6, *A–D*) and minor differences in the microbial composition were observed (Fig. 6, *E*–*G*) when comparing co-housed lean and obese mice. Of note, only Ruminococcus_1 was discriminatory for the ob/ob mice, whereas Lachnospiraceae_ASF356 was discriminatory for the lean mice. Yet, as the mucus phenotype was not different between the two co-housed genotypes, we conclude that these taxa are not the drivers of the mucus defect observed in genetically obese mice but are rather associated with their lean/obese phenotype.

**Figure 7.**
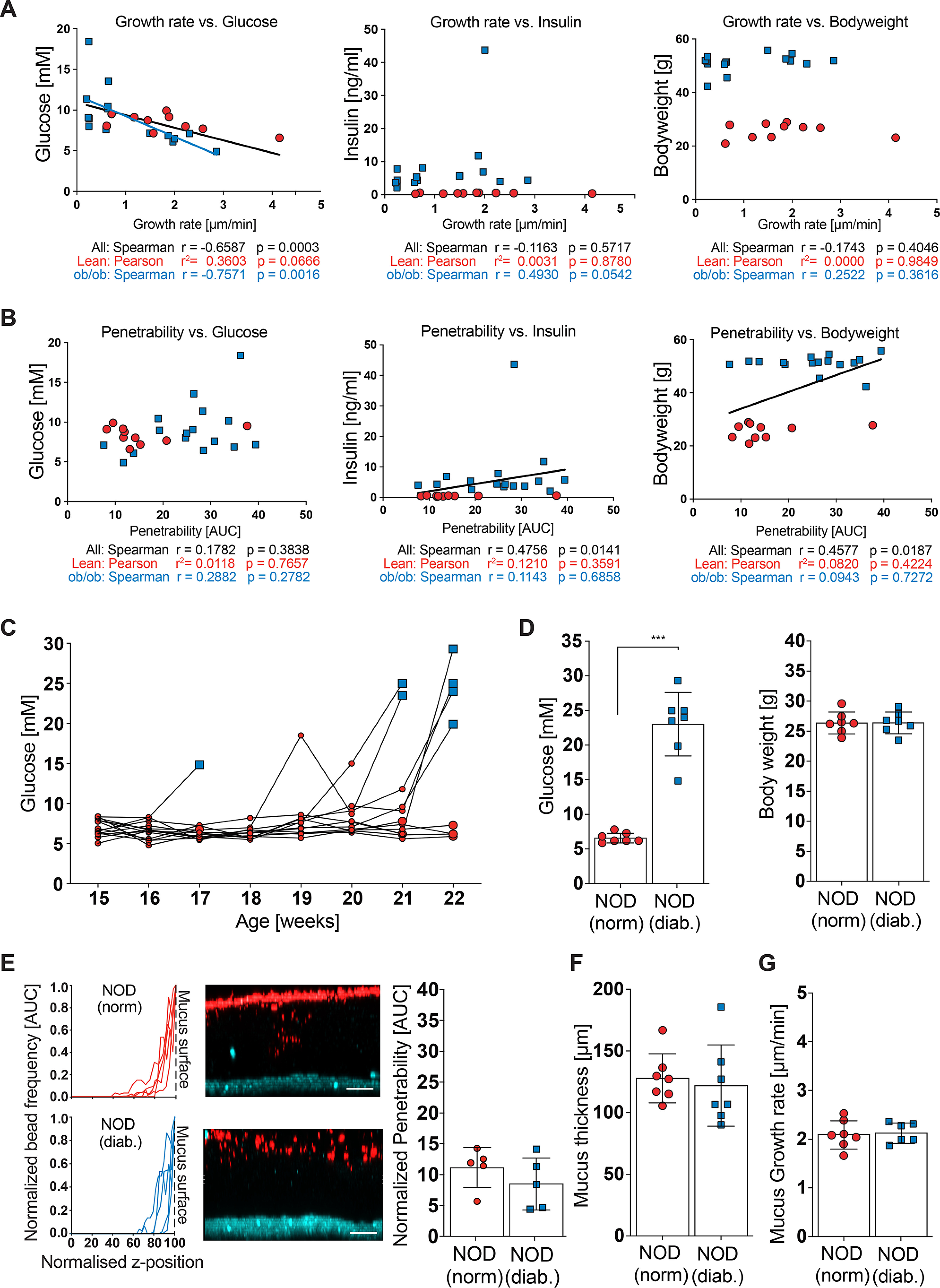
**Mucus function and glucose metabolism.** *A* and *B*, mucus growth rate (*A*) and mucus penetrability (*B*) were correlated with fasting blood glucose (*left*), blood insulin concentration (*center*) and body weight (*right*) (*n* = 10-15 mice per genotype). Pearson (normally distributed data) or Spearman (not-normally distributed data) correlation analysis was used to test correlations between metabolic phenotypes and mucus function. A *black regression line* indicates significant correlation with all samples, whereas a *blue line* indicates significant correlation within the ob/ob group. *C*, post-prandial glucose levels of normoglycemic (*red*) and diabetic (*blue*) NOD mice were measured weekly. Time points of sacrifice/mucus measurement have enlarged symbols. *D*, post-prandial glucose levels and body weight of normoglycemic (*norm*.) and diabetic (*diab*.) NOD mice before mucus analyses (*n* = 7 mice per genotype). *E*, mucus properties in the distal colon. From left to right: the position of fluorescent 1-μm beads, obtained from confocal z-stacks, were used to determine penetrability of the inner colonic mucus layer. *Turquoise*, intestinal tissue; *red*, 1 μm bacteria-sized beads. *Scale bar* = 50 μm. *F*, thickness and growth rate (*G*) of the mucus layer was measured *ex vivo* with a micromanipulator by measuring the distance between black 10-μm beads and the epithelial surface (*right*) (*n* = 7 mice per group). Data in *D–G* are presented as mean ± S.D. *, *p* ≤ 0.05; **, *p* ≤ 0.01; ***, *p* ≤ 0.001 (Mann-Whitney *U* test). *E*, for the normalized bead frequency (*left*), the median bead frequency distribution is shown for each mouse.

As we observed differences in glucose levels between the genotype-separated and co-housed mice we aimed to test whether the mucus defect in genetically obese mice might be driven by glucose concentration. We therefore correlated mucus growth rate and penetrability with glucose and insulin concentration as well as body weight. Indeed, we observed a significant correlation between fasting blood glucose concentration and mucus growth rate (*p* = 0.0003), which was also significant when the ob/ob group was analyzed individually (*p* = 0.0016) (Fig. 7*A*). No correlation was observed between mucus growth rate and insulin levels (*p* = 0.5717) or body weight (*p* = 0.4046). Mucus penetrability, in contrast, did not correlate with fasting blood glucose concentration (*p* = 0.3838), but instead correlated with insulin concentration (*p* = 0.0141) and body weight (*p* = 0.0187) (Fig. 7*B*). However, these latter correlations did not hold when testing within-group correlations, and we thus conclude that those overall correlations are rather due to the comparison of dissimilar groups, as indicated by the distribution of the obese (*blue squares*) and lean (*red circles*) mice. Also, body weight and insulin levels are comparable between the co-housed and genotype-separated mouse sets, which further indicates that neither of these factors is a primary contributor to the mucus defect.

To further investigate if glucose levels could contribute to altered mucus phenotypes we determined mucus function in non-obese diabetic (NOD) mice, which develop spontaneous type-1 diabetes with strongly increased blood glucose levels but without developing obesity. As diabetes onset is spontaneous (Fig. 7*C*) and dependent on genetic and environmental conditions (23), we co-housed NOD mice and compared age- and weight-matched diabetic mice with their normoglycemic cage mates (Fig. 7*D*). When mucus function was analyzed in these mice, no differences in mucus penetrability (Fig. 7*E*) nor mucus thickness (Fig. 7*F*) or growth rate (Fig. 7*G*) were observed; both mouse groups had a healthy, functioning inner colonic mucus layer. We thus conclude that the observed mucus defect in genetically obese mice is independent of blood glucose levels.

## Discussion

Intestinal mucus function is mainly studied during dietary interventions, and a defective mucus layer is observed after consumption of high-fat and/or fiber-free diets in mice (4, 5, 7). Here we show that a defect of the colonic mucus can also occur under a standard dietary regimen in pre-diabetic, genetically obese mice. Although the quality of the diet is the same between lean and ob/ob mice, the quantity differs tremendously and might be a confounding factor contributing to the defective mucus. However, because co-housing of ob/ob and lean littermates abrogated the obesity-induced mucus phenotype but not the obesity-associated metabolic phenotypes, including increased food intake and altered motility, this strongly suggests the microbiota as the mucus-modulating factor in ob/ob mice. Although the gut microbiota composition between lean and obese mice was not tremendously different, relative abundance of *Akkermansia* was lower in the colon of ob/ob mice (Fig. 2*D*). This is in agreement with previous studies that linked reduced abundance of *Akkermansia muciniphila* to obesity (24) and that demonstrated that a purified membrane protein from *A. muciniphila* could improve metabolism in obese and diabetic mice (25). Moreover, application of the anti-diabetic drug metformin to mice slightly increased the number of mucus-producing goblet cells in the small intestine, an effect that was paralleled by increased relative abundance of *A. muciniphila* (26), which is consistent with reduced relative abundance of *Akkermansia* in obese mice that have reduced mucus production (24). However, when investigating mucus function *ex vivo*, relative abundance of *Akkermansia* did neither correlate with mucus growth rate (*p* = 0.9507; *r* = −*0*.0234) nor with mucus penetrability (*p* = 0.1571; *r* = −0.4166), so we conclude that the abundance of *Akkermansia* alone is likely not sufficient to maintain intact mucus function.

A family that was highly represented in the discriminatory ASVs for genotype-separated ob/ob mice was the *Ruminococcaceae* (Fig. 2*E*). A member of this family, *Ruminococcus gnavus* has been shown to be transiently enriched in patients with inflammatory bowel disease (27, 28) and with pre-diabetes (29), who have defects in intestinal barrier function, including their mucus layer (11, 30, 31). Moreover, one *R. gnavus* strain has been shown to degrade mucus glycans *in vitro* (32). Yet, in our analyses we did not reveal a role of *R. gnavus* in mucus penetrability of the ob/ob mice.

We identified several (unclassified) ASVs of the Lachnospiraceae and Muribaculaceae families that were discriminatory for the lean as well as for the ob/ob mice. Accordingly, our findings highlight that either additional low abundant bacteria may be responsible for the mucus phenotype or that the function of the microbial ecosystem is altered in obesity (33, 34). Support for the latter hypothesis could arise from a previous study (3), in which microbial communities from two different mouse colonies in the same animal facility had differential effects on mucus function, primarily mucus penetrability. Similarly, no causal relationship between individual gut microbiota members and mucus properties were identified, yet demonstrated the transfer of the gut microbial communities into germ-free mice clearly that the gut microbiota was causative regarding the mucus phenotype.

Our microbiota diversity analyses indicated a significant clustering according to genotype when lean and ob/ob mice were separated (Figs. 2 and 3). Still, we also observed a degree of intra-group variability within the lean and obese groups. This variability can at least in part be explained by the inclusion of more than one litter into the experimental groups. Of note, the influence of kinship on the microbiota diversity was much stronger in the co-housed groups than in the genotype-separated groups, demonstrating that the microbiota exchange through co-housing was successful.

Recently, we identified *Bifidobacterium longum* as a mucus-function modulating bacterium in mice (7). The abundance of *Bifidobacterium* was reduced in WSD-fed mice and supplementation with *B. longum* or the *Bifidobacterium*-promoting dietary fiber inulin could prevent the mucus defects. However, in this study we could not identify a specific depletion of *Bifidobacterium*, indicating that a more complex interaction with the residual microbial community probably contributes to its beneficial effect on mucus function.

Our current and previous results (7) describe a defect of the colonic mucus layer in genetically and diet-induced obesity in mice. In agreement, metabolic diseases have been linked to defects in the intestinal mucosal barrier, and it has been shown that translocation of microbial products, such as lipopolysaccharide, across the epithelial barrier can initiate obesity and insulin resistance (8, 9). Moreover, bacterial penetration into the colonic mucus layer was observed in patients with insulin resistance–associated dysglycemia, but not in healthy controls (11). However, from these observations it is not clear whether microbial mucus penetration is a cause or consequence of the metabolic impairments. Support for the hypothesis that barrier dysfunction is secondary to metabolic impairments has been obtained from a comprehensive study that included several mouse models of obesity and glucose metabolism (35). The authors found that hyperglycemia promotes intestinal barrier dysfunction through GLUT2-dependent transcriptional reprogramming of intestinal epithelial cells, which was followed by alteration of tight and adherence junction integrity (35). To test whether increased glucose concentration can also promote the mucus defect, we analyzed mucus function in diabetic NOD mice. Remarkably, these mice had a healthy, functioning inner colonic mucus layer, indicating that glucose metabolism does not cause a mucus defect. This observation is in contrast to a recent investigation, in which the authors concluded that abnormalities in mucus function in NOD mice precede the diabetes development (36). However, in that study NOD mice and their controls were obtained from different providers and mucus production was interpreted from counting goblet cells in suboptimal fixed tissue. As the microbial community often differs between mouse providers (37) and the intestinal microbiota are contributing to diabetes development in NOD mice (38, 39), that analysis rather shows a microbiota-dependent effect than linking mucus dysfunction directly to glucose metabolism.

In summary, we conclude that the mucus defect in genetically obese mice is microbiota dependent, but that the responsible taxa needs to be identified. However, it is likely that the overall composition of the complex community, including their metabolites, rather than individual taxa affects mucus function in leptin-deficient mice. Moreover, and based on our *ex vivo* methodology to study mucus function, we furthermore, conclude that functional impairment of the inner colonic mucus layer is no prerequisite to induce diabetes in mice. However, as we studied mucus function in NOD mice already a few days after their glucose levels raised, we cannot exclude that chronically increased blood glucose levels may lead to mucus defects in the colon. It thus requires more investigations in mice and humans to disentangle the complex interaction between the intestinal mucus barrier and metabolic diseases, such as obesity and insulin resistance.

## Experimental procedures

### Mice

Mice were co-housed with up to 5 mice/cage under specific pathogen-free conditions at a 12-h light/dark cycle and had unlimited access to water and food. All mouse experiments were approved by the University of Gothenburg Animal Studies Committee. Leptin-deficient ob/ob mice on a C57Bl/6J background (B6.Cg-*Lep^ob^*/J) were originally obtained as heterozygous pairs from Charles River (Germany), bred in-house and either co-housed with their lean littermates or separated according to genotype after weaning, often resulting in lower number of mice per cage. All mice were genotyped and the “lean” mouse group includes WT (wt/wt) and 14–60% heterozygous (wt/ob) mice, which did not differ in metabolic parameter or microbiota β-diversity when compared with wt/wt mice (*q* between 0.156 and 1.00). The experimental groups contain litters from three breeding pairs, explaining the variation in some of the groups.

Female NOD mice (NOD/ShiLtJ strain) were obtained from The Jackson Laboratory (Italy) and were co-housed with 5 mice/cage. All mice used were age matched (15-17 weeks old for ob/ob set; 17-22 weeks for NOD mice) and fed a standard chow diet (5021 LabDiet: 4.62 kcal/g; 23.7% kcal from fat (31% saturated, 33% monounsaturated, 36% polyunsaturated fatty acid), 53.2% from carbohydrates (sucrose 0.71% (w/v), starch 31.0% (w/v) glucose 0.21% (w/v), neutral detergent fiber 15.2% (w/v)). Animals were anesthetized using isofluorane and killed by cervical dislocation prior to sample collection. Sampling time during the day was matched between groups. For NOD mice, body weight and post-prandial glucose levels were determined once a week and mice with increased blood glucose levels (>14.5 mm) were weight-matched with a nondiabetic NOD mouse as a control for mucus analysis.

### Metabolic measurements

Blood glucose concentration was measured postprandial (NOD mice) or after fasting for 4 h (ob/ob) in tail vein whole blood with commercial blood glucose strips (Contour Next, Bayer, Germany). Insulin concentration in ob/ob mice was determined in serum by Ultra-Sensitive Mouse Insulin ELISA kit (Crystal Chem Inc., IL).

### Explant ex vivo mucus thickness measurements

Thickness of the mucus layer was measured on live tissue as described (15). Briefly, intestinal tissue was flushed with cold Krebs buffer to remove luminal content and unattached mucus. The muscle layer was removed by microdissection and the intestine was mounted in a horizontal chamber system and maintained at 37 °C with basolateral Krebs-glucose buffer perfusion and apical Krebs-mannitol buffer. For visualization of the mucus surface black 10-μm polystyrene Polybead microspheres (Polysciences, Germany) were added apically. The surface was observed by a stereomicroscope and mucus thickness measured using a glass micropipette connected to a micrometer. For the inner colonic mucus layer, mucus thickness was measured every 15 min at five different locations for up to 45 min to calculate the mucus growth rate.

### Confocal analysis of mucus layer properties

Mucus function was assessed by quantifying penetrability to 1-μm bacteria-sized microbeads by use of confocal microscopy. Flushed intestinal tissue was prepared and mounted in a horizontal chamber as previously described (15). The tissue was stained by adding calcein violet AM (Thermo Fisher Scientific, MA; 1 μg/ml) to the basolateral Krebs/glucose buffer and the apical mucosal surface was overlaid with 1-μm diameter Fluosphere crimson microbeads (Thermo Fisher, 1:10) diluted in 10 μl of Krebs/mannitol buffer. Microbeads were allowed to sediment onto the mucus surface for 5 min and the mucosal surface was gently washed with 0.5 ml of Krebs/mannitol to remove excess microbeads. The apical chamber compartment was then filled with 2 ml of Krebs/mannitol and the perfusion chamber was transferred to an LSM700 confocal imaging system (Carl Zeiss, Germany). Tissue and microbeads were visualized by acquiring confocal z-stacks using a ×20 water immersion objective lens, 405/639 nm lasers, and Zen acquisition software (Carl Zeiss). Zen files were imported into Imaris software (Bitplane) and isosurfaces were mapped to calcein violet (tissue) and Fluosphere (microbeads) fluorescent signals. Data describing the *z* axis position of the tissue and individual microbeads was extracted and the mucus layer thickness was quantified by calculating the average tissue-microbead *z* axis distance. Mucus penetrability was quantified by analysis of microbead distribution within the mucus layer. Initially, a frequency distribution curve of the tissue-microbead *z* axis distance data were generated for each z-stack using Prism 7 software (GraphPad). To allow comparison of distribution curves acquired from different z-stacks, curves were first normalized to maximum frequency values to correct for differences in the absolute number of microbeads detected in each z-stack. Subsequently curves were normalized to the position of the mucus surface (the *z* axis position with the maximum microbead frequency) to correct for variable mucus layer thickness, and were cropped to exclude data from microbeads above the mucus surface. Area under the curve data were generated for each normalized distribution curve and expressed as “normalized penetrability” to allow quantitative comparison of microbead penetration into the mucus layers of different samples.

### DNA extraction and 16S rRNA gene sequencing

Genomic DNA from mucosal tissue, and intestinal content was extracted by repeated bead-beating using a Fast-Prep System with Lysing Matrix E (MPBio, CA) as described previously (40). Bacterial DNA present in luminal content and intestinal tissue was profiled by sequencing of the V4 region of the 16S rRNA gene on an Illumina MiSeq (Illumina RTA version 1.17.28; MCS version 2.5) using 515F and 806R primers designed for dual indexing (41) and the V2 kit (2 × 250 bp paired-end reads). Intestinal content samples were amplified in duplicates, whereas mucosa-associated bacteria (MAB) samples were amplified in triplicates in reaction volumes of 25 μl containing 1× Five Prime Hot Master Mix (Quantabio, MA), 200 nm of each primer, 0.4 mg/ml of BSA, 5% DMSO, and 20 (content samples) or 100 ng (MAB) of genomic DNA. PCR was carried out under the following conditions: initial denaturation for 3 min at 94 °C, followed by 25 cycles (content samples) or 28 cycles (MAB samples) of denaturation for 45 s at 94 °C, annealing for 60 s at 52 °C, and elongation for 90 s at 72 °C, and a final elongation step for 10 min at 72 °C. Replicates were combined, purified with the NucleoSpin Gel and PCR Clean-up kit (Macherey-Nagel, Germany) and quantified using the Quant-iT PicoGreen dsDNA kit (Thermo Fisher Scientific). Equal amounts of purified PCR products were pooled and the pooled PCR products were purified again using Ampure magnetic purification beads (Agencourt, Danvers, MA) to remove short amplification products.

Illumina reads were merged using Usearch version 11 64-bit (42), allowing for up to 30 mismatches (43) in the alignment of the paired-end reads, whereas discarding reads with a merged length greater than 270 bp and less than 230 bp. The merged reads were quality filtered based on expected errors, removing reads above the threshold of 1 (44). The merged reads were turned into zero-radius operational taxonomic units (Zotus) (45) by compiling the sequences into sets of unique reads and performing error-correction using the UNOISE3 algorithm (46), discarding sequences with fewer than 4 reads. The Zotus were assigned taxonomy using DADA2's (47) assignTaxonomy (minBoot = 50) and assignSpecies, using a formatted version of the Silva version 132 database. A phylogenetic tree of the sequences was created with the help of the MAFFT software version 7.407 (48) and the FastTree software version 2.1.10. Very low abundant sequences (relative abundance <0.002%) were excluded from the analysis. To correct for differences in sequencing depth (median = 53,813; range 7110–102431) for diversity analyses, the same amount of sequences were randomly subsampled for each group of samples (rarefaction; 35,300 reads for luminal content, 7,000 reads for mucosa-associated bacteria). The software package QIIME II (version 2020.2) (49) was used to compute α- and β-diversity of the samples as well as relative abundance of individual taxa. Statistical differences between groups were calculated by using Kruskal-Wallis test (α-diversity) or PERMANOVA and 999 permutations (β-diversity).

Wald test implemented in DESeq2 was used in differential abundance analyses for all count data and raw *p* values were adjusted by the Benjamini–Hochberg method (50) with a false discovery rate of 5%. Heatmap was constructed based on hierarchical clustering of the differentially abundant ASVs using spearman correlations.

### Quantification of fermentation products

Caecal short-chain fatty acids were measured using GC coupled to MS detection (GC-MS). Approximately 20-100 mg of caecal content were mixed with internal standards, added to glass vials and freeze dried. All samples were then acidified with HCl, and SCFAs were extracted with two rounds of diethyl ether extraction. The organic supernatant was collected, the derivatization agent *N*-tert-butyldimethylsilyl-*N*-methyltrifluoroacetamide (Sigma-Aldrich) was added and samples were incubated at room temperature overnight. SCFAs were quantified with a gas chromatograph (Agilent Technologies 7890A) coupled to a mass spectrometer (Agilent Technologies 5975C).

### Statistical analyses

Statistical analyses have been carried out with GraphPad Prism (version 8.4) if not stated otherwise under “Experimental procedures.” For comparisons between two groups an unpaired *t* test has been used when samples were distributed normally. For not normally distributed samples Mann-Whitney *U* test has been used. In all figures data are presented as mean ± S.D. For correlation analysis normality was tested with D'Agostino and Pearson normality test and in the case of normal distribution, Pearson correlation coefficients were calculated; for not normally distributed data nonparametric Spearman correlation was tested. A linear regression line in the figure indicates a significant correlation. Statistical analyses and visualization for differential microbial abundances for 16S rDNA have been performed with QIIME 2 (version 2020.2)(49) and R (version 3.5.1; 2018-07-02); differences in α-diversity have been tested with Kruskal Wallis test, whereas β-diversity has been analyzed with PERMANOVA and 999 permutations.

## Data availability

Microbiota 16S rDNA gene sequencing results have been deposited in the European Nucleotide Archive with accession number PRJEB35863.

## Supplementary Material

Supporting Information
