# Supplementary material for: Obesity-associated microbiota contributes to mucus layer defects in genetically obese mice
Source: J Biol Chem. 2020 Sep 8;295(46):15712–26. doi: 10.1074/jbc.RA120.015771 (PMC7667970; doi:10.1074/jbc.RA120.015771)
Supplement: Supporting Information [file supp_295_46_15712__index.html]

Obesity-associated microbiota contributes to mucus layer defects in genetically obese mice — Mucus defect in genetically obese mice — Obesity-associated microbiota contributes to mucus layer defects in genetically obese mice — Mucus defect in genetically obese mice — Supporting Information 

# Obesity-associated microbiota contributes to mucus layer defects in genetically obese mice

## Supporting Information

- Supporting Information (to be published online) - Supplementary Figures 1+2
