# Supplementary material for: Obesity-associated microbiota contributes to mucus layer defects in genetically obese mice
Source: J Biol Chem. 2020 Sep 8;295(46):15712–26. doi: 10.1074/jbc.RA120.015771 (PMC7667970; doi:10.1074/jbc.RA120.015771)
Supplement: Supporting Information [file supp_RA120.015771_163118_1_supp_592413_qgd01t.pdf]

Obesity-associated microbiota contributes to mucus layer defects in genetically obese mice

Bjoern O. Schroeder[1,2]*, George M.H. Birchenough[3], Meenakshi Pradhan[1], Elisabeth E.L. Nyström[3], Marcus Henricsson[1], Gunnar C. Hansson[3] and Fredrik Bäckhed[1,4,5]

Supporting information contains Supplementary Figures S1-S2

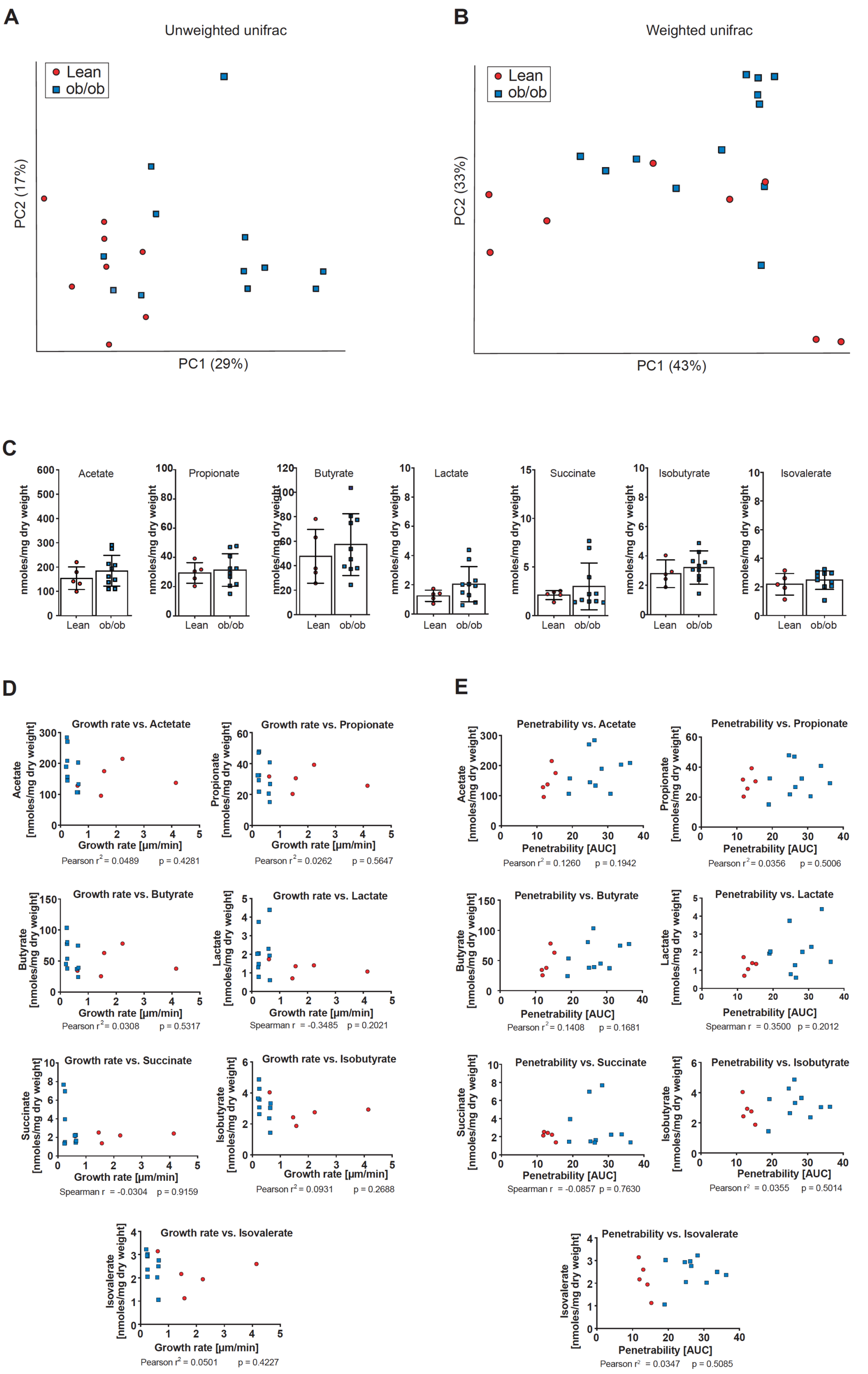

Figure S1 – Gut microbiota diversity and fermentation products in the intestinal lumen of genotype-separated ob/ob mice.
A, **B** Beta-diversity of lean and ob/ob mice. Principal component analysis plots using unweighted (A) and weighted (B) unifrac distance matrices are shown. (n = 8-12 mice per genotype). Beta-diversity was calculated with PERMANOVA and 999 permutations. **C** Microbial fermentation products were measured in the caecal content of ob/ob and lean mice (n = 5-10 mice per genotype). Data are presented as mean ± SEM. *P≤0.05 (Mann-Whitney U test). **D, E** Microbial fermentation products were correlated with mucus growth rate (D) and mucus penetrability (E) of the inner colonic mucus layer. Correlation analyses were performed with Pearson test for normally distributed data and with Spearman rank test for not-normally distributed data.

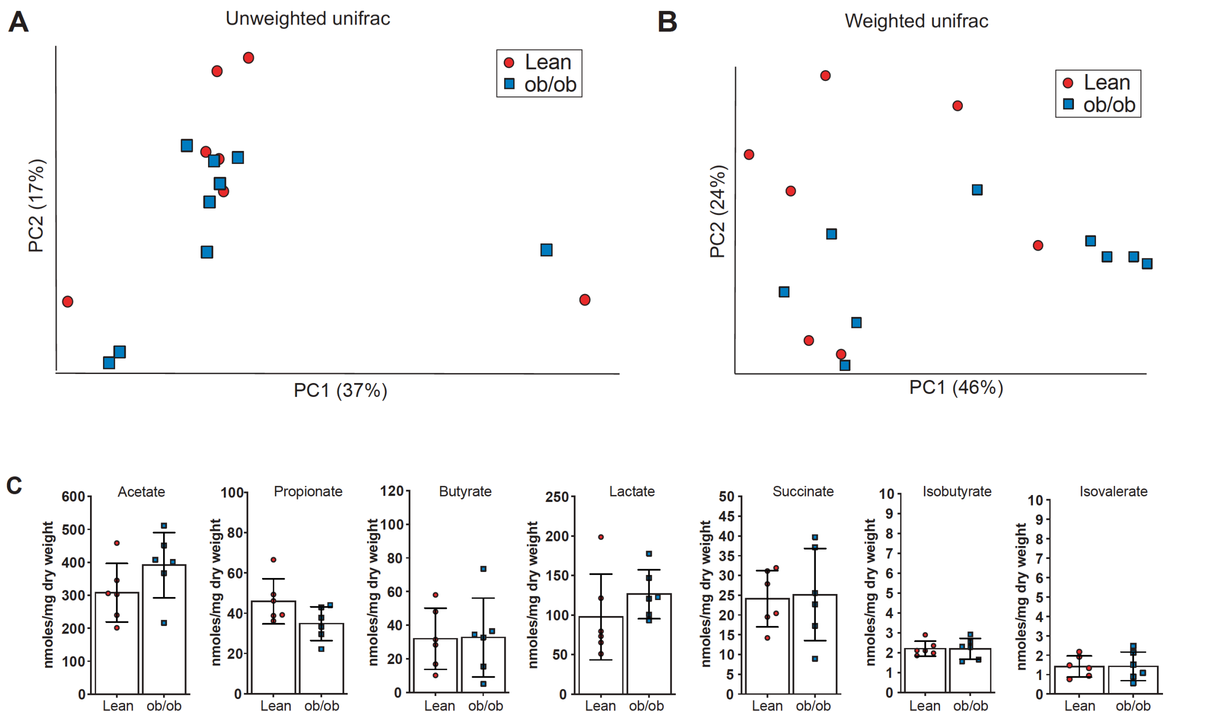

Figure S2 – Gut microbiota diversity and fermentation products in the intestinal lumen of co-housed ob/ob mice.

A, **B** Beta diversity of co-housed lean and ob/ob mice. Principal component analysis plots using unweighted (A) and weighted (B) unifrac distance matrices are shown. (n = 7-9 mice per genotype). Beta-diversity was calculated with PERMANOVA and 999 permutations. **C** Microbial fermentation products were measured in the caecal content of ob/ob and lean mice (n = 6 mice per genotype). Data are presented as mean ± SD. *P≤0.05 (Mann-Whitney U test).
